# Positive and Negative Regulation of Th17 Cell Differentiation: Evaluating The Impact of *RORC2*

**Published:** 2014-10-04

**Authors:** Mazdak Ganjalikhani Hakemi, Kamran Ghaedi, Vida Homayouni, Alireza Andalib, Mohsen Hosseini, Abbas Rezaei

**Affiliations:** 1 Cellular and Molecular Immunology Research Center, Isfahan University of Medical Sciences, Isfahan, Iran; 2 Department of Immunology, Faculty of Medicine, Isfahan University of Medical Sciences, Isfahan, Iran; 3 Department of Biology, Faculty of Sciences, University of Isfahan, Isfahan, Iran; 4 Department of Epidemiology, Faculty of Health, Isfahan University of Medical Sciences, Isfahan, Iran; 5 Applied Physiology Research Center, Isfahan University of Medical Sciences, Isfahan, Iran

**Keywords:** *IL-17*, *IL-23R*, *RORC2*, siRNA, Th17

## Abstract

**Objective:**

Th17 cells are known to be involved in some types of inflammations and autoimmune disorders. *RORC2* is the key transcription factor coordinating Th17 cell differentiation. Thus, blocking *RORC2* may be useful in suppressing Th17-dependent inflammatory processes. The aim was to silence *RORC2* by specific siRNAs in naïve T cells differentiating to Th17. Time-dependent expression of *RORC2* as well as *IL-17* and *IL-23R* were considered before and after *RORC2* silencing.

**Materials and Methods:**

In this experimental study, naïve CD4^+^T cells were isolated from human cord blood samples. Cytokines TGFß plus IL-6 and IL-23 were used to polarize the naïve T cells to Th17 cells in X-VIVO 15 serum free medium. A mixture of three siRNAs specific for *RORC2* was applied for blocking its expression. *RORC2*, *IL-17* and *IL-23R* mRNA and protein levels were measured using qRT-PCR, ELISA and flow cytometry techniques. Pearson correlation and one-way ANOVA were used for statistical analyses.

**Results:**

Significant correlations were obtained in time-dependent analysis of *IL-17* and *IL-23R* expression in relation with *RORC2* (R=0.87 and 0.89 respectively, p<0.05). Silencing of *RORC2* was accompanied with almost complete suppression of *IL-17* (99.3%; p<0.05) and significant decrease in *IL-23R* gene expression (77.2%, p<0.05).

**Conclusion:**

Our results showed that *RORC2* is the main and the primary trigger for upregulation of *IL-17* and *IL-23R* genes in human Th17 cell differentiation. Moreover, we show that day 3 could be considered as the key day in the Th17 differentiation process.

## Introduction

*IL-17*-producing helper T cells (Th17) are identified as a new subtype distinct from other types of T cells (1, 2). The discovery of the Th17 cell and its biological functions improved our understanding of the roles of helper T cells in adaptive immunity and disease pathogenesis (3, 4). Human Th17 cells express high levels of *IL-23R*, IL-1R1 and IL-18Rα as well as CCR6 and CCR4 on their surface (5, 6). Th17 cells induce production of chemokines and anti-microbial peptides by tissue cells which causes the recruitment of neutrophils into tissues and induces inflammation (5-11). In addition, Th17 cells are associated with pathogenesis of several inflammatory and autoimmune diseases such as multiple sclerosis, rheumatoid arthritis, psoriasis, inflammatory bowel disease and periodontitis (3, 4, 8, 12-15), a role previously as signed to Th1 and IFN-γ (6, 8, 12). Hence, there is a great interest to study the molecular aspects of its differentiation and regulation, which may lead to the development of new efficient approaches for regulation of inflammation caused by these cells.

It is believed that retinoic acid-related orphan nuclear receptor-C2 (*RORC2*) is the key transcription factor which coordinates the Th17 cell differentiation and its over-expression induces *IL-17* production (5-8, 12, 16-19). Thus, silencing *RORC2* gene expression could be helpful in inhibiting the polarization of human naïve CD4^+^ T cells to Th17 cells. Accordingly, it is speculated that gene silencing methods for *RORC2* inhibition may be utilized as a potential therapeutic target for treatment of Th17-dependent inflammatory diseases.

The aim of the present study was to silence the *RORC2* gene by specific siRNAs. The effect of this silencing was also evaluated on other Th17 characteristic genes, including *IL-17* and *IL-23R*. Time-dependent expression pattern of Th17 characteristic genes was also considered to find the level of gene expression before and after targeting the *RORC2* gene by siRNA transfection.

## Materials and Methods

The ethical aspects of this experimental study were approved by the Ethics Committee of Isfahan University of Medical Sciences, Isfahan, Iran.

### Purification of naive CD4^+^ T cells

Cord blood samples were taken from umbilical cord of newborns in Shahid Beheshti Hospital, Isfahan, Iran. Mononuclear cells were separated from 100 ml cord blood sample using Ficoll-Hypaque density gradient method (Biosera, France). Naive CD4^+^ T cells were isolated using the human naive CD4^+^ T cell isolation kit II (Miltenyi Biotech, Germany) according to manufacturer's instruction as follows: in brief, CD45RO^+^ activated/memory T cells and non- CD4^+^ T cells were magnetically labeled and depleted with a cocktail of biotin-conjugated antibodies against CD8, CD14, CD15, CD16, CD19, CD25, CD34, CD36, CD45RO, CD56, CD123, TCRγ/δ, HLA-DR, CD235a (Glycophorine A) and anti-biotin micro-beads (2; 8; 13). Isolation of highly pure naive CD4 T cells was confirmed by flow cytometry after immune staining with FITC conjugated anti-CD4 and PE conjugated anti-CD45RA antibodies (BD Biosciences, San Jose, USA). Cell analysis was performed with FACSCalibur and data were analyzed with CellQuest-Pro software (BD Biosciences, San Jose, USA).

### Cell culture and differentiation assay

Each well of 48-well plates (Orange, Belgium) was treated by 100 μl PBS including 5μg/ml anti-CD3 antibody and 2μg/ml anti- CD28 antibody (eBiosciences, USA) and incubated at 4˚C overnight. Naïve CD4^+^ T cells were then cultured in these plates at a density of 1×10^5^ cells per well in X-VIVO 15 serum free medium (Lonza, Swiss) treated with TGF-β (10 ng/ml), IL-23 (100 ng/ml), IL-6 (30 ng/ml), anti-IFN-γ (10 μg/ml) and anti-IL-4 (10 ng/ml) (eBioscience, USA). The culture media and all the components were refreshed after 3 days. On the sixth day, the cells were washed and their viability was checked by trypan blue exclusion (2, 20, 21).

### Cell transfection with siRNA

Three siRNA oligonucleotides specific for different positions on *RORC2* mRNA were previously designed (Table 1) (22) and T cells were transfected with a mixture of these siRNAs on the third day, using TransIT-TKO Transfection Reagent (Mirus, USA) as instructed by the manufacturer. For 3-5×10^5^ cells per well, 4 μl TransIT-TKO Transfection Reagent, 50 nM of siRNA (final concentration) and 50 μl of serumfree medium OptiMEM were added. Untransfected T cells were used as control. In order to exclude siRNA and/or transfection toxicity, T cells transfected with scrambled siRNA and T cells treated with transfection reagent without siRNA (mock control) were used as toxicity controls. The cells were incubated overnight and then, medium and all of its contents (except for the transfection polyplex) were refreshed. Transfection efficiency in CD4^+^ T cells was confirmed using flow cytometry after transfecting cells with Label IT® RNAi Delivery Control kit (Mirus, USA).

**Table 1:** The specific siRNAs sequences against RORC2 gene


	siRNA name	Start position	siRNA sequence

**1**	OptiRNA	872	5´-CCUCCCUGACAGAGAUAGATT-3´ 3´-TTGGAGGGACUGUCUCUAUCU-5´
**2**	siDirect	1197	5´-CCGCACGGUCUUUUUUGAATT-3´ 3´-TTGGCGUGCCAGAAAAAACUU-5´
**3**	Ambion	1393	5´-GUAGAACAGCUGCAGUACATT-3´ 3´-TTCAUCUUGUCGACGUCAUGU-5´


### Cell viability test

Metabolic activity of transfected T cells was evaluated by methylthiazole tetrazolium (MTT) assay and is briefly as follows: 10 μL of a 5 mg/ mL MTT solution in PBS buffer (Sigma-Aldrich, Germany) was added to each well of the 96-well plate. After 1h of incubation at 37˚C and 5% CO_2_, the medium was removed and T cell-containing plates were frozen for 1 hour at -80˚C. Afterwards, the purple formazan product was dissolved in 100 μL/well dimethyl sulfoxide (DMSO) (Sigma- Aldrich, Germany) at 37˚C for 30 minutes while shaking. Optical density was quantified by a microplate reader (ELX 800) at 590 nm (reference wavelength 630 nm), and viability of the cells was reported as a percentage compared with untrasfected control cells.

### RNA isolation, cDNA synthesis and quantitative RT-PCR

Total RNA from cultured CD4^+^ T cells was extracted using RNeasy mini kit (Qiagen, USA) and cDNA was synthesized using QuantiTect reverse transcription kit (Qiagen, USA) as instructed by the manufacturer. The resulting transcripts were then quantified by real time quantitative PCR on a Step One Plus TM real time DNA amplification system (Appiled Biosystems, USA) with Quanti- Fast SYBR Green PCR kit (Qiagen, USA). Pre-designed primers (QuantiTact primer Assay; Qiagen, USA) specific for *IL-17*, *IL-23R* and *RORC2* were used. For each sample, transcript quantity was normalized to the amount of beta-actin (ACTB) expression. The obtained results were analyzed by the relative quantification method (23, 24).

### Measurement of cytokine concentration

Cytokine contents of supernatant culture media were measured with an *IL-17* ELISA kit (RayBiotech, Norcross, GA) according to the manufacturer’s instruction. Results were read by a microplate reader (ELX 800) at 450 nm.

### Flow cytometric analysis

CD4^+^ T cells were collected from culture plates on the sixth day. The cells were first stained extracellularly with phycoerythrin (PE)-labeled anti- *IL-23R* antibody (R&D Systems, USA). Then, the cells were fixed and permeabilized with BD Cytofix/Cytoperm Plus (BD Bioscience, USA) and subsequently were stained intracellularly with peridinin chlorophyll protein complex (PerCP)- conjugated anti-*RORC2* antibody (R&D Systems, USA). After incubation, the samples were acquired on a FACSCalibur instrument (BD Biosciences, San Jose, USA) and data were analyzed with CellQuest-Pro software (BD Biosciences, San Jose, USA).

### Statistical analysis

The Pearson correlation coefficient test was used to evaluate the level of correlations and their significance among studied markers of Th17 cells. P-values less than 0.05 were considered statistically significant. One-way ANOVA is used for comparison between control groups and cells which were treated with siRNAs. All experiments were carried out in triplicate and data are presented as mean and standard deviation (SD) in graphs. All the above was performed using SPSS 16.0 software (Chicago, USA).

## Results

### Time dependent expression pattern of Th17 characteristic genes

**Fig 1:**
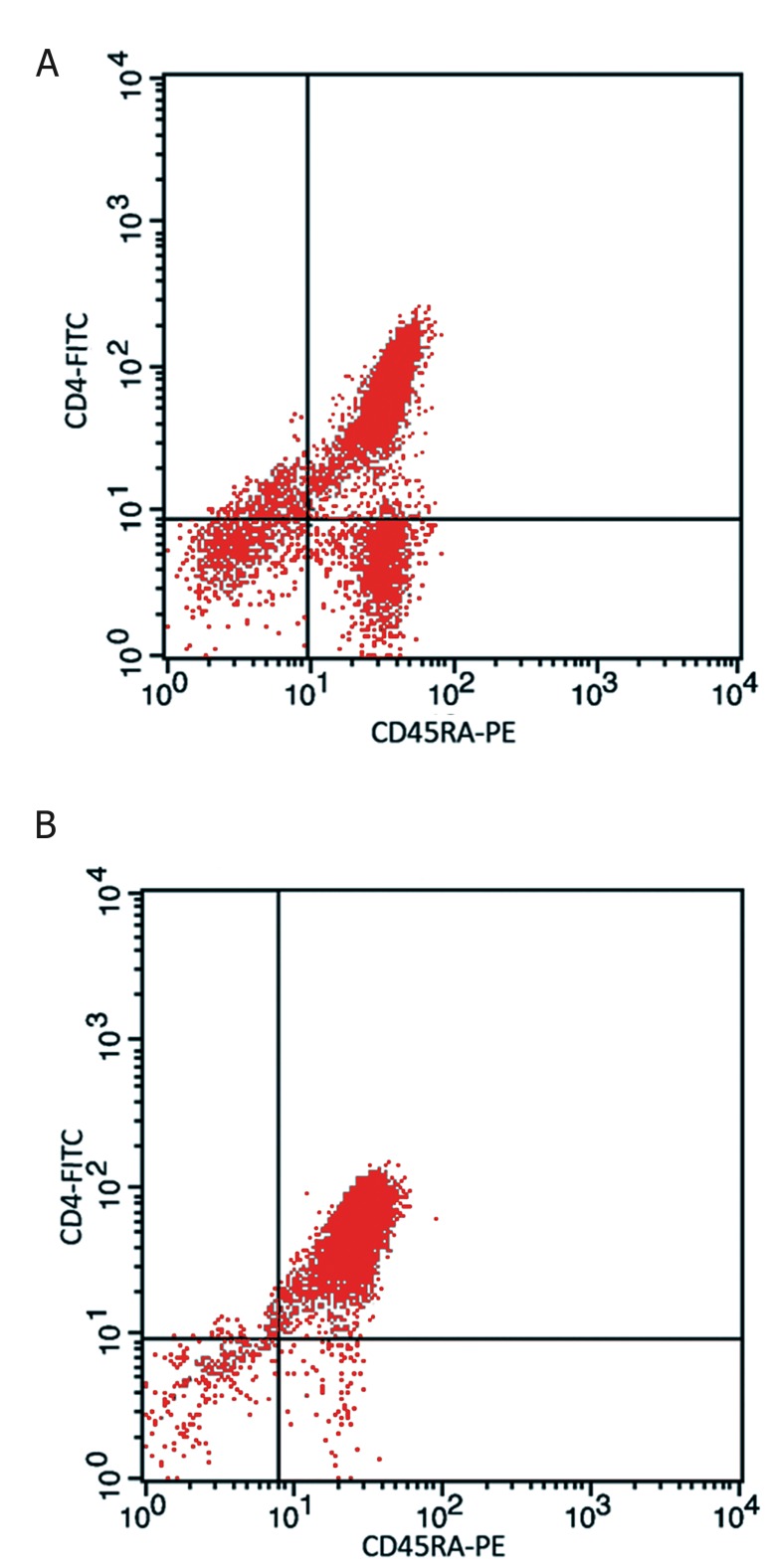
Flow cytometric analysis of naïve CD4^+^ T cell subsets isolated from cord blood samples. A. Dot plot diagram shows three distinct cell populations in cord blood mononuclear cells separated via ficoll hypaque density gradient, before the isolation of human naïve CD4^+^ T cells. B. The diagram shows more than 95% purity of CD4^+^/CD45RA^+^ T cells in elution flow samples obtained from human naïve CD4^+^ T cell isolation column.

Highly pure naïve T cells were isolated from cord blood samples. Based on flow-cytometric analysis, more than 95% of isolated cells were CD4^+^/CD45RA^+^ cells which represent naïve T cells (Fig 1).

**Fig 2:**
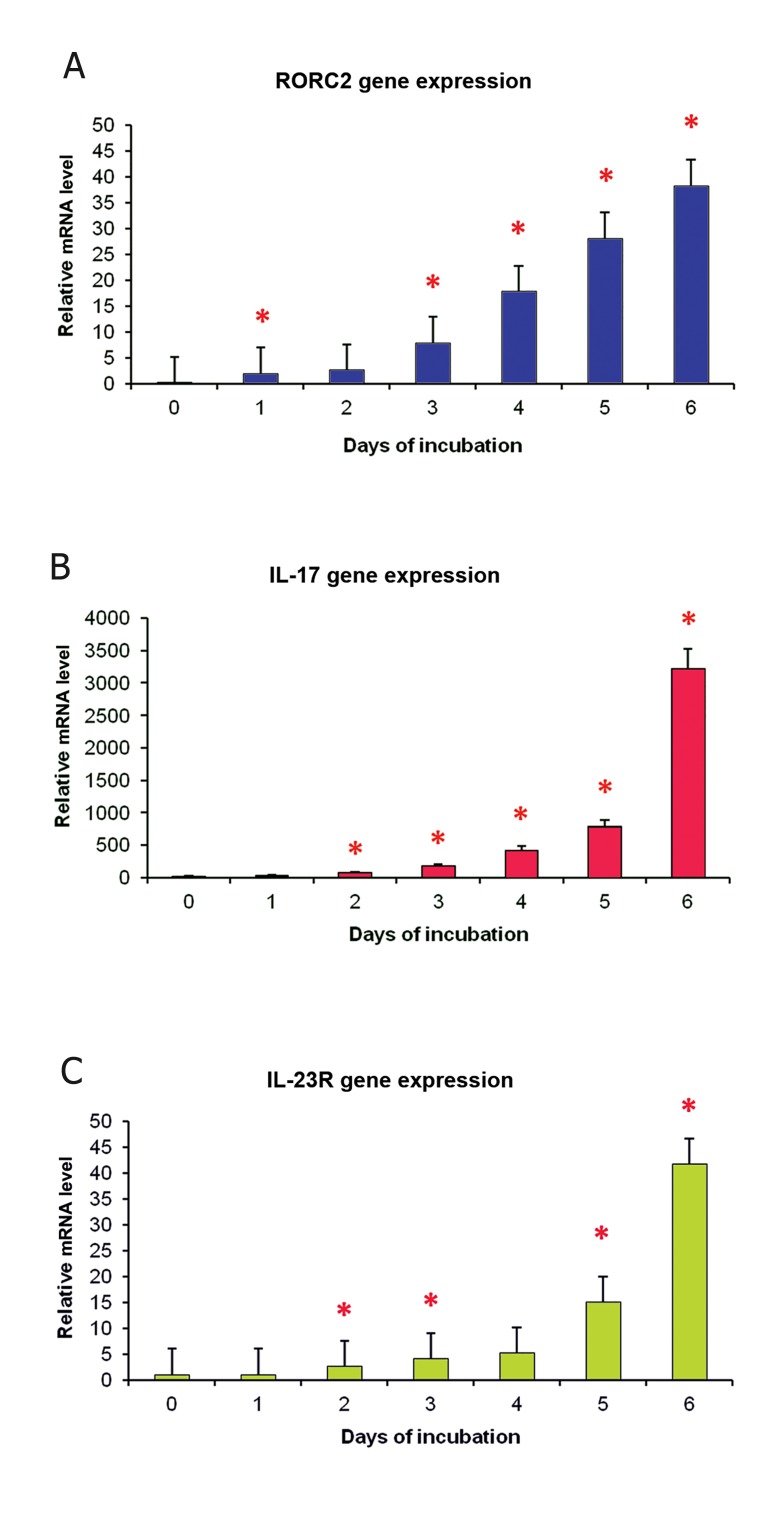
Time dependent expression study for *RORC2*, *IL-17* and *IL-23R* genes during Th17 differentiation using quantitative RT-PCR. Transcript levels of selected genes were measured each day after T cells were cultured in condition polarizing towards Th17 cells. A. *RORC2* gene expression significantly elevated from the 1st day of culture and after a slight increase, sharply rose on day 3 (p<0.05). B. *IL-17* gene expression was increasingly elevating starting from the 1st day of culture, but a significant increase level appeared at day 2 (p<0.05). C. *IL-23R* gene expression was increasingly elevating starting from the 2nd day of culture, (p<0.05). Asterisks show significant elevation of mRNA level in comparison with the previous day. Data are shown with relative unit and are the mean and SD of three identical experiments.

A time course analysis for expression of *RORC2* gene was performed during 6-day culture of T cells polarizing to Th17 cells. Cell proliferation began after the second day and viability of the cells was more than 98% based on trypan blue exclusion test. During the 6-day culture, every day a sample was taken and evaluated for *RORC2* gene expression. Figure 2A shows that *RORC2* transcript levels were incrementally elevated from the first day of incubation to day 6 with an ascent in day 3 (expression was 3 times more on the third day compared with the second day; p<0.05).

Simultaneous with *RORC2*, the expression level of the other Th17 characteristic genes, *IL-17* and *IL-23R*, were also analyzed and similar results were obtained. As indicated in figures 2B and 2C, the expression levels of these genes were duplicated in the second day of culture. This was statistically significant compared with days 0 and 1 (p<0.05).

A significant correlation was observed among the expression patterns of *RORC2*, *IL-17* and *IL-23R* genes during Th17 cell differentiation (Table 2).

**Table 2:** Correlations between RORC2, IL-17 and IL-23R gene expression during Th17 cells differentiation


		IL-17	IL-23R

RORC2	Pearson correlation	0.87^*^	0.89^*^
	Sig. (2-tailed)	0.024	0.015
	N	18	18


### The Effect of *RORC2* knock down on *IL-17* and *IL-23R* expression

**Fig 3:**
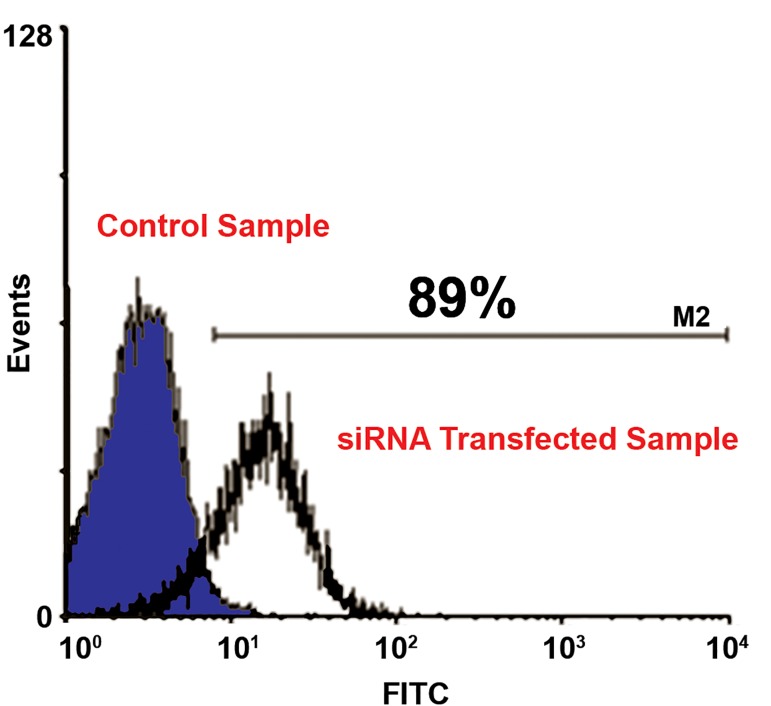
Human CD4^+^ T cells 24 hours after transfection with flourescein-labeled siRNA. The flow-cytometric histogram indicates 89% transfection efficiency in transfected T cells. Gray curve represents untransfected cells.

**Fig 4:**
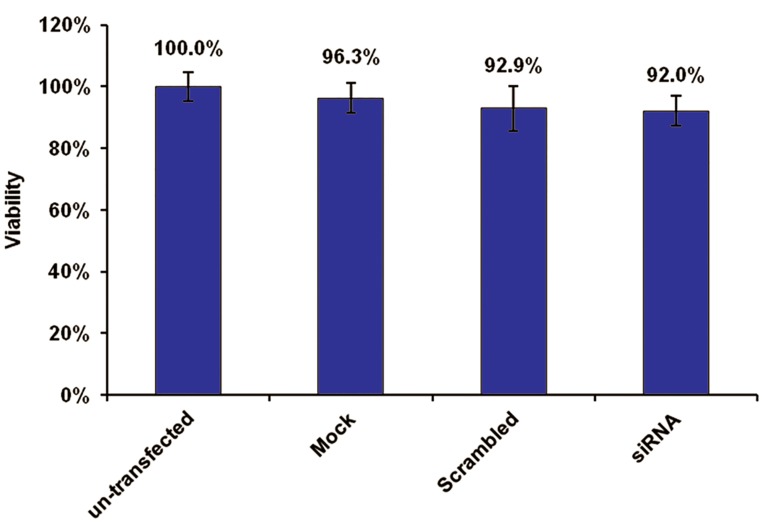
Cell viability testing of CD4^+^ T cells using MTT assay. Compared with untreated control cells, 92% of siRNA transfected (test) cells were alive. Viability in scrambled siRNA transfected cells was almost similar to test cells (92.9%). No significant variation in cell viability was seen among the test and control wells.

Following transfection of naïve T cells, transfection efficiency was quantitatively evaluated by flow cytometry (Fig 3). Transfection efficiency was 89% with no significant toxicity in any sample. After transfection, cell viability was 92% compared with untreated control cells based on MTT assay (Fig 4). Cell viability was confirmed by trypan blue staining.

Based on time-dependent expression study, we transfected the T cells with a cocktail of siRNAs specific for *RORC2* on day 3. One night after transfection, culture medium was refreshed and the polarizing procedure carried out.

**Fig 5:**
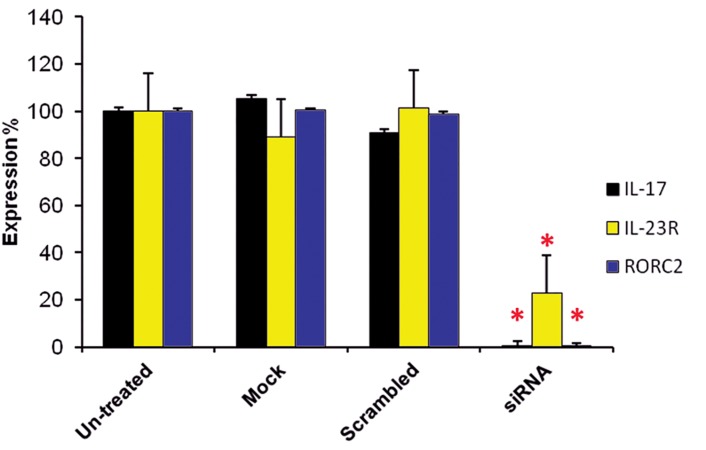
Measurement of *RORC2*, *IL-17* and *IL-23R* transcript levels by qRT-PCR following *RORC2* gene suppression. *RORC2* was significantly suppressed using specific siRNAs in cultured T cells (* p<0.05). Silencing of *RORC2* led to a significant suppression in *IL-17* and *IL-23R* gene expression (* p<0.05). Each experiment was performed in triplicate. Data are shown in percentage scale and are the mean and S.D of five identical experiments. Control (untreated): neither transfection reagent nor siRNA; Mock control (mock): no siRNA; control siRNA (Scrambled): using Label IT® RNAi Delivery Control.

**Fig 6:**
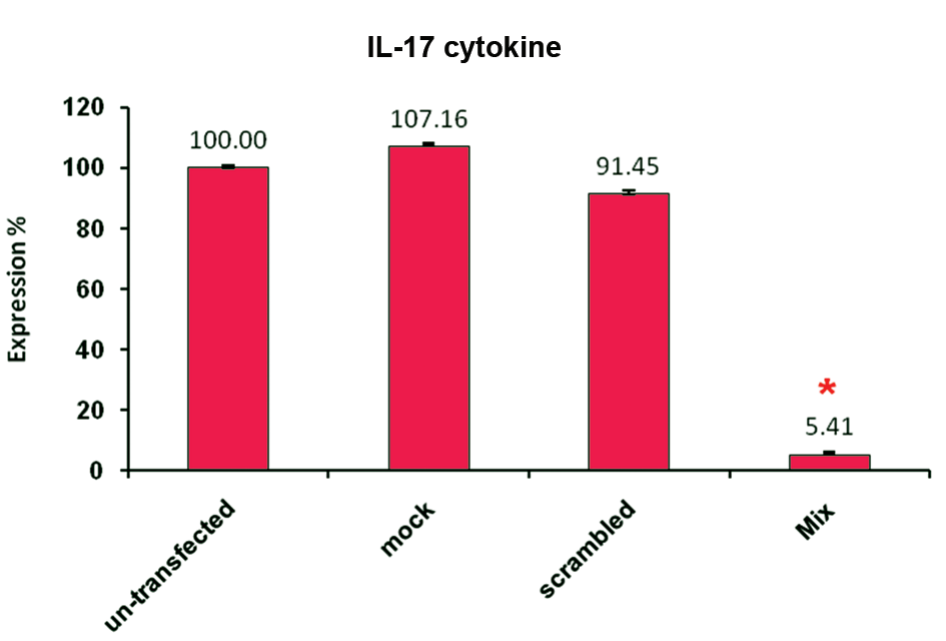
Measurement of *IL-17* cytokine by ELISA following *RORC2* gene suppression. *IL-17* protein production was significantly suppressed after transfection of cultured T cells with *RORC2* specific siRNAs (* p<0.05). Each experiment was performed in triplicate. Data are shown in percentage scale and are the mean and S.D of three similar experiments. Control (untreated): neither transfection reagent nor siRNA; Mock control (mock): no siRNA; control siRNA (Scrambled): using Label IT® RNAi Delivery Control.

On the sixth day, qRT-PCR was performed and a significant suppression (99.5%) in *RORC2* gene expression was observed in comparison with untransfected T cells, whereas no significant effect was obtained with scrambled siRNA which confirms the specificity of the assay (Fig 5). Simultaneously, the transcript level of *IL-17* in polarizing T cells, whose *RORC2* expression was blocked, showed 99.3% inhibition (p<0.05) (Fig 5). In addition, there was a significant correlation between *RORC2* and *IL-17* expression (R=0.99; p=0.000) following *RORC2* suppression. Similarly, *IL-17* cytokine production was reduced to 5.41% in comparison with untransfected control cells (Fig 6).

As seen in Figure 5, siRNA mediated suppression of *RORC2* reduced the level of IL-23 receptor mRNA by 77.2% in the T cells (p<0.05). The correlation between *IL-23R* expression and *RORC2* gene suppression status was equal to 0.65 with p=0.001.

**Fig 7:**
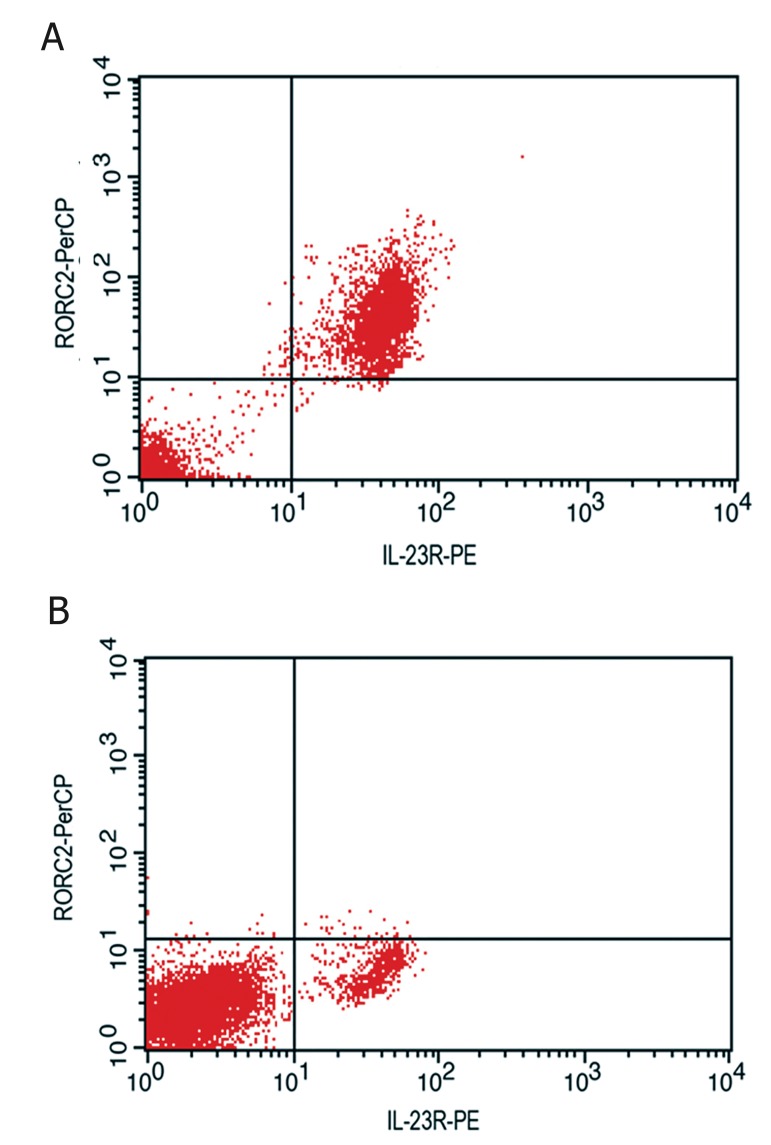
Flow cytometric analysis of *RORC2* and *IL-23R* expression in polarized Th17 cells before and after silencing of *RORC2* gene. All analyses were performed on R1 gated cells from a homogenous population. A. On day 6 of differentiation, the cells were harvested and stained with anti-*RORC2*-PerCP mAb (yaxis), then fixed, permeablized and stained with *IL-23R*–PE mAb (x-axis), and analysed by flow cytometry. Percentage in upper-right quadrant shows that 88.6% of polarized T cells are *RORC2*^+^/*IL-23R*^+^ cells. B. After silencing of *RORC2* with specific siRNA, the dot plot diagram shows significant decrease in *RORC2*^+^/*IL-23R*^+^ cell population. There are still 29.3% IL- 23R^+^ cells in lower-right quadrant of the dot plot diagram after *RORC2* specific siRNA transfection.

Flow cytometric analysis of the siRNA tranfected cell population also confirmed that the percentage of *RORC2*^+^/*IL-23R*^+^ cells was only 1.9% in comparison with untransfected T cells (p<0.05). However, 29.3% of the cells were still positive for IL-23 receptor (Fig 7).

## Discussion

In the history of medicine, scientists have strived to overcome diseases more effectively through developing new therapeutic methods. Since the discovery of RNAi in 1998, significant efforts and resources have been invested in exploiting its therapeutic applications (25, 26). The original therapeutic indications for siRNA have been performed *in vivo* using viral strains (e.g. HIV, hepatitis B and C, respiratory syncytial virus, poliovirus and herpes simplex virus) and cancer models (a wide variety of mutated oncogenes such as K-Ras, mutated p53, Her2/ neu, and bcr-abl) (27, 28). This approach has been recently applied for treatment of various diseases (8, 27).

Based on the important role of Th17 cells in autoimmune disorders, it is believed that *RORC2* gene could be one of the main transcription factors in Th17 cell development (7, 16, 17, 29). It is therefore speculated that post-transcriptional suppression of *RORC2* gene expression could be potentially a promising therapeutic approach for these types of diseases.

In the present study, we used naive CD4^+^ T cells isolated from human cord blood. These were cultured in an optimized condition preparing for Th17 cell development (21). In six day study of a culture of CD4^+^ T cells, examining Th17 characteristic genes (*RORC2*, *IL-17* and *IL-23R* genes) revealed an almost simultaneous and strongly correlated increase in their mRNA levels, starting within 24 hours after stimulation for *RORC2* and within 48 hours for *IL-17* and *IL-23R*. Ivanov II et al. have reported an elevation of RORƒÁt mRNA level at 16 hours and for *IL-17* at 48 hours after stimulation of Th17 polarization (7). Ichiyama K et al. (30) have also reported that the elevation of RORγt mRNA level starts within 24 hours of the formation of the culture leading to a polarizing condition toward Th17 cells.

Although these studies have been carried out using murine Th17 cells, our results are consistent with them. This confirms the importance of *RORC2* for regulation of *IL-17* and *IL-23R* expression in human Th17 cells. However, our literature search did not display any results on *RORC2* and *IL-23R* time-dependent expression patterns in human T lymphocytes for comparison.

Significant correlation among *RORC2*, *IL-17* and *IL-23R* gene expression was observed during the duration of the experiments. However, in the present study, these correlations (Table 2) seem to be stronger than previous studies (2, 21, 30). This might be due to the application of the single most optimized condition for Th17 polarization in the current study.

It has been reported that in cytokine-induced Th17 cells, *IL-17* expression is significantly increased following *RORC2* up-regulation (31). Although they have used different polarization processes, their results confirm our findings. Moreover, our work covers more aspects of differentiation regulation such as the effect of *RORC2* up-regulation on *IL-23R* expression as well as the effect of specific *RORC2* silencing on *IL-17* expression.

Further observations revealed an increase of the *RORC2* gene expression on day 3 which was followed by a similar increase for *IL-17* and *IL-23R* on day 4 (Fig 2) and 5 (Fig 3) respectively. This indicates that *IL-23R* is expressed at a slower rate, compared with the expression of *IL-17* gene during Th17 differentiation. A review of the literature found no such report to compare with this finding. Based on these observations, day 3 can be considered as the key day in commitment of naïve CD4^+^ T cells in differentiating into Th17 lineage and hence, *RORC2* silencing occurred on the third day.

According to the Ichiyama’s study, IL-6 suppresses the Foxp3 expression in differentiating T cells within 48-72 hours which can stimulate more *RORC2* expression (30). As IL-6 was one of the polarizing components to Th17 cells in the present study, these results are consistent with our findings.

In the current study, our data revealed an almost complete suppression of *RORC2* gene expression at 50 nM final concentration of specific siRNA, while cells maintained a high metabolic activity. The mRNA and protein level of *IL-17* were severely decreased following siRNA mediated decrease of *RORC2*, which means a significant positive correlation between them.

A similar point was mentioned by Burgler et al. in murine model (32). In addition, Volpe et al. (2) have reported that even a decrease of about 50% in *RORC2* mRNA expression is sufficient to inhibit *IL-17* expression. In our study, the stronger silencing effect of *RORC2* might be attributable to the difference in the sequences of the siRNAs which we have designed and other properties of the shRNA which they have used. However, the nature of the shRNA in their study was not clarified for comparison. Nevertheless, in both studies, silencing of *RORC2* was followed by dramatic decrease in *IL-17* expression.

We show that the effect of *RORC2* on *IL-17* expression is more when it is suppressed (Table 3). This suggests that, although transcription factors other than *RORC2* are probably participating in *IL-17* up-regulation (2, 4, 21, 29, 30), *RORC2* can be considered as the main and primary trigger for that, as its suppression leads to almost complete down-regulation of *IL-17* expression.

**Table 3:** Correlations between RORC2, IL-17 and IL-23R gene expression in CD4^+^ T cells after silencing of RORC2 gene using specific siRNA


		IL-17	IL-23R

RORC2	Pearson correlation	0.99^**^	0.65^*^
	Sig. (2-tailed)	0.000	0.001
	N	5	5


Genetic and flow cytometric analyses also revealed a significant drop in *IL-23R* expression following *RORC2* inhibition. Although a significant correlation was observed, it was not as much as what we obtained in *RORC2* upregulation (Table 3). In addition, this effect on *IL-23R* was less than what had been observed for *IL-17* gene expression. We did not find any report in the literature for comparative analysis.

It is documented that signaling of *IL-23R* via JAK2/STAT3 activates *RORC2* gene expression which in turn, up-regulates *IL-17* gene. *RORC2* and *IL-17* gene expression are necessary for expansion and maintenance of Th17 phenotype which is reflected by more *IL-23R* expression (4, 33). Therefore, down-regulation of *RORC2* which inhibits *IL-17* gene expression diminishes the expression of *IL-23R* on T cells. On the other hand, *IL-23R* signaling through NF-κB up-regulates *IL-17* expression (33) and hence, decreased *IL-23R* results in down-regulation of *IL-17* gene. Consequently, *IL-17* is suppressed both directly via *RORC2* knock down and indirectly via *IL-23R* down-regulation. Thus, we suggest more detailed studies to clarify the exact molecular mechanism of *IL-17* and *IL-23R* gene expression regulation through *RORC2* action.

## Conclusion

The results of the current study suggest that suppression of *RORC2* expression could be an efficient barrier for human Th17 polarization pathway. Therefore, *RORC2* can be considered as an important therapeutic target for Th17 cell development inhibition in inflammatory disorders. Such a therapeutic aim may be achieved by either using *RORC2* specific siRNA accompanied by a suitable *in vivo* delivery system or by applying the appropriate pharmacological antagonists for *RORC2* gene function. Therefore, we recommend further *in vitro* and animal model studies to evaluate the effect of *RORC2* suppression on any autoimmune or inflammatory disease associated with Th17 cells.

## References

[1] Cardoso CR, Garlet GP, Crippa GE, Rosa AL, Junior WM, Rossi MA (2009). Evidence of the presence of T helper type 17 cells in chronic lesions of human periodontal disease. Oral Microbiol Immunol.

[2] Volpe E, Servant N, Zollinger R, Bogiatzi SI, Hupe P, Barillot E (2008). A critical function for transforming growth factor-beta, interleukin 23 and proinflammatory cytokines in driving and modulating human T(H)-17 responses. Nat Immunol.

[3] Ouyang W, Kolls JK, Zheng Y (2008). The biological functions of T helper 17 cell effector cytokines in inflammation. Immunity.

[4] McGeachy MJ, Cua DJ (2008). Th17 cell differentiation: the long and winding road. Immunity.

[5] Zhu J, Paul WE (2008). CD4 T cells: fates, functions, and faults. Blood.

[6] Streeck H, Cohen KW, Jolin JS, Brockman MA, Meier A, Power KA (2008). Rapid ex vivo isolation and long-term culture of human Th17 cells. J Immunol Methods.

[7] Ivanov II, McKenzie BS, Zhou L, Tadokoro CE, Lepelley A, Lafaille JJ (2006). The orphan nuclear receptor RORgammat directs the differentiation program of proinflammatory IL-17^+^ T helper cells. Cell.

[8] Acosta-Rodriguez EV, Napolitani G, Lanzavecchia A, Sallusto F (2007). Interleukins 1beta and 6 but not transforming growth factor-beta are essential for the differentiation of interleukin 17-producing human T helper cells. Nat Immunol.

[9] Aggarwal S, Ghilardi N, Xie MH, de Sauvage FJ, Gurney AL (2003). Interleukin-23 promotes a distinct CD4 T cell activation state characterized by the production of interleukin-17. J Biol Chem.

[10] Ghilardi N, Ouyang W (2007). Targeting the development and effector functions of TH17 cells. Semin Immunol.

[11] Park H, Li Z, Yang XO, Chang SH, Nurieva R, Wang YH (2005). A distinct lineage of CD4 T cells regulates tissue inflammation by producing interleukin 17. Nat Immunol.

[12] Wilson NJ, Boniface K, Chan JR, McKenzie BS, Blumenschein WM, Mattson JD (2007). Development, cytokine profile and function of human interleukin 17-producing helper T cells. Nat Immunol.

[13] Yang L, Anderson DE, Baecher-Allan C, Hastings WD, Bettelli E, Oukka M (2008). IL-21 and TGF-beta are required for differentiation of human T(H)17 cells. Nature.

[14] Harrington LE, Hatton RD, Mangan PR, Turner H, Murphy TL, Murphy KM (2005). Interleukin 17-producing CD4^+^ effector T cells develop via a lineage distinct from the T helper type 1 and 2 lineages. Nat Immunol.

[15] Adibrad M, Deyhimi P, Ganjalikhani Hakemi M, Behfarnia P, Shahabuei M, Rafiee L (2012). Signs of the presence of Th17 cells in chronic periodontal disease. J Periodontal Res.

[16] Aranami T, Yamamura T (2008). Th17 cells and autoimmune encephalomyelitis (EAE/MS). Allergol Int.

[17] Gocke AR, Cravens PD, Ben LH, Hussain RZ, Northrop SC, Racke MK (2007). T-bet regulates the fate of Th1 and Th17 lymphocytes in autoimmunity. J Immunol.

[18] Laurence A, Tato CM, Davidson TS, Kanno Y, Chen Z, Yao Z (2007). Interleukin-2 signaling via STAT5 constrains T helper 17 cell generation. Immunity.

[19] Oboki K, Ohno T, Saito H, Nakae S (2008). Th17 and allergy. Allergol Int.

[20] Vyakarnam A, Vyas B, Vukmanovic-Stejic M, Gorak- Stolinska P, Wallace D, Noble A, Rowland Jones SL, Andrew JM (2000). Human CD4 culture. Lymphocytes and a practical approach.

[21] Ganjalikhani Hakemi M, Ghaedi K, Andalib A, Hosseini M, Rezaei A (2011). Optimization of human Th17 cell differentiation in vitro: evaluating different polarizing factors. In Vitro Cell Dev Biol Anim.

[22] Ganjalikhani Hakemi M, Ghaedi K, Andalib A, Homayouni V, Hosseini M, Rezaei A (2013). RORC2 gene silencing in human Th17 cells by siRNA: design and evaluation of highly efficient siRNA. Avicenna J Med Biotechnol.

[23] Livak KJ, Schmittgen TD (2001). Analysis of relative gene expression data using real-time quantitative PCR and the 2(-Delta Delta C(T)) method. Methods.

[24] Pfaffl MW (2001). A new mathematical model for relative quantification in real-time RT-PCR. Nucleic Acids Res.

[25] Frohlich T, Wagner E (2010). Peptide- and polymer-based delivery of therapeutic RNA. Soft Matter.

[26] Thomas M, Greil J, Heidenreich O (2006). Targeting leukemic fusion proteins with small interfering RNAs: recent advances and therapeutic potentials. Acta Pharmacol Sin.

[27] Ichim TE, Li M, Qian H, Popov IA, Rycerz K, Zheng X (2004). RNA interference: a potent tool for genespecific therapeutics. Am J Transplant.

[28] Dallas A, Vlassov AV (2006). RNAi: a novel antisense technology and its therapeutic potential. Med Sci Monit.

[29] Yang XO, Pappu BP, Nurieva R, Akimzhanov A, Kang HS, Chung Y (2008). T helper 17 lineage differentiation is programmed by orphan nuclear receptors ROR alpha and ROR gamma. Immunity.

[30] Ichiyama K, Yoshida H, Wakabayashi Y, Chinen T, Saeki K, Nakaya M (2008). Foxp3 inhibits RORgammat- mediated IL-17A mRNA transcription through direct interaction with RORgammat. J Biol Chem.

[31] Crome SQ, Wang AY, Kang CY, Levings MK (2009). The role of retinoic acid-related orphan receptor vari ant 2 and IL-17 in the development and function of human CD4^+^ T cells. Eur J Immunol.

[32] Burgler S, Mantel PY, Bassin C, Ouaked N, Akdis CA, Schmidt-Weber CB (2010). RORC2 is involved in T cell polarization through interaction with the FOXP3 promoter. J Immunol.

[33] Cho ML, Kang JW, Moon YM, Nam HJ, Jhun JY, Heo SB (2006). STAT3 and NF-kappaB signal pathway is required for IL-23-mediated IL-17 production in spontaneous arthritis animal model IL-1 receptor antagonist-deficient mice. J Immunol.

